# Quetiapine‐induced chest wall edema with the swellings of face and extremities in a young hospitalized patient: A case report

**DOI:** 10.1002/ccr3.7661

**Published:** 2023-07-18

**Authors:** Forouzan Elyasi, Fatemeh Heydari, Kaveh Haddadi

**Affiliations:** ^1^ Sexual and Reproductive Health Research Center, Psychiatry and Behavioral Sciences Research Center, Addiction Institute Mazandaran University of Medical Sciences Sari Iran; ^2^ Department of Psychiatry, Faculty of Medicine Mazandaran University of Medical Sciences Sari Iran; ^3^ Department of Anesthesiology, Faculty of Medicine Mazandaran University of Medical Sciences Sari Iran; ^4^ Orthopedic Research Center Mazandaran University of Medical Sciences Sari Iran

**Keywords:** adverse effect, antipsychotics, case report, quetiapine

## Abstract

**Key Clinical Message:**

Quetiapine can lead to the face, extremity and particularly chest wall edema in hospitalized patients in the supine position.

**Abstract:**

Quetiapine (QTP) is known as an atypical antipsychotic agent with some adverse effects, such as edema. However, along this line, peripheral edema is not a life‐threatening episode, but it is an important side effect affecting medical compliance. Therefore, QTP‐induced chest wall edema with the swellings of the face and the extremities is very rare. This report is about a young man who was admitted in the intensive care unit with multiple trauma (MT). On account of his delirious state, QTP was started at 25 mg and then increased to 75 mg, three times a day. The patient developed swelling of the face, the upper and lower limbs, and the chest wall. After stopping the QTP use, his edema went down. Although there is still speculation about the possible mechanisms of antipsychotic‐induced edema, some studies have pointed to the relationship between dopaminergic antagonism and peripheral edema. Therefore, it is very important to pay close attention to this side effect.

## 1 INTRODUCTION

Quetiapine (QTP) is known as an atypical antipsychotic agent with a high affinity for 5‐hydroxytryptophan (5‐HTP) and D2 dopamine receptors.^1^ One of the therapeutic applications of QTP is the treatment of delirium, as a neuropsychiatric syndrome with fluctuations in the course of the disease, disturbances of consciousness, disorientation, reduced awareness of surroundings and attention deficit.^2^ This condition also known as the most common diagnosis of the central nervous system (CNS) involvement and is common in hospitalized patients, particularly those admitted in the intensive care units (ICUs).^3^ In this sense, edema is a symptom of tissue swellings caused by an increase in the volume of interstitial fluid. Local edema in the hands, wrists, feets and knees is called peripheral edema, which usually occurs either as a result of a systemic disease (e.g. liver cirrhosis, kidney disease, congestive heart failure, lymphedema, and hypoproteinemia) or cancers and even by substances such as nonsteroidal anti‐inflammatory drugs (NSAIDs), antihypertensives, immunosuppressants and steroids.^4^ Although peripheral edema is not a life‐threatening event, it is a significant adverse event that impacts treatment compliance.^5^ There have been several reports about peripheral edema caused by QTP.^6^, ^7^, ^8^ In this regard, Polat et al.^5^ reported five cases of QTP‐induced peripheral edema. Two cases have also been reported in two studies about facial and eyelid edema ‐in addition bilateral lower extremity edema.^9^, ^10^ To the authors' knowledge, there have been no reports of chest wall edema along with swellings of the face and the extremities following the use of QTP. This case report accordingly aims to introduce a young man, admitted to an ICU, suffering from this complication after the administration of QTP.

## 2 CASE PRESENTATION

The patient was a 20‐year‐old young man with an opioid use disorder (OUD) who suffered from falling down and paraplegia while serving in the military. He underwent surgery for a fractured thoracic vertebra. After multiple trauma (MT), the patient experienced fractures of T4, T5, and T6 vertebrae, along with an open wound on the back with exudate. After developing a fever, he was transferred from another center to a university general hospital in one of Iran's northern cities. Following the surgical site infection (SSI), the device was removed and the fever subsided. As well, he was intubated and connected to a respiratory ventilator after severe pulmonary empyema, and then, a bilateral chest tube was installed. The patient also received a short‐acting morphine sulfate infusion for 10 days that followed by 10 mg of intramuscular methadone 10 mg, twice a day. Because of his restlessness, the consultation‐liaison psychiatry services (CLPS) team visited the patient on two consecutive days and 4 days later in 2018. The first visit occurred 6 days after hospitalization. At this point, the patient was intubated and restless, and he removed the angiocatheter. According to the Diagnostic and Statistical Manual of Mental Disorders, Fifth Edition (DSM‐5) criteria^11^, ^12^ and the Confusion Assessment Method for the ICU (CAM‐ICU) as the diagnostic algorithm, opium use disorder (OUD) and delirium were diagnosed. Later, severe SSI was suggested as the main cause of delirium. Some related investigations, including blood cultures and cultures of infection at the surgical site, were also performed, and patient was monitored regularly by a neurosurgeon, a critical care specialist and an infectious disease specialist and received the appropriate antibiotics. QTP was started at 12/5 mg, three times a day, and increased to 25 mg, three times a day on the second day after delirium due to continued agitation. Of note, the dosage was increased to 50 mg three times a day on the sixth day and then to 75 mg three times a day (225 mg daily) on the ninth day after the start of the QTP for agitation due to hyperactive delirium. On the sixth day after the QTP administration, an ultrasound sonography was performed on the patient due to scrotal swelling and afterward consultation with a urologist was ordered. The ultrasound report showed a bilateral increase in scrotal thickness along with many tracts of liquid, indicating scrotal edema. During the consultation with the urologist, the mechanism of injury was suggested as the cause the edema, and scrotal elevation was recommended. As well, 15 days after the psychiatric consultation for delirium, the patient was consulted once again due to depressed mood. Until then, he continued to receive 225 mg QTP/day for persistent delirium. At this visit, the patient was conscious and oriented and the delirium had subsided. The patient presented the giving up‐given up complex, hopelessness and helplessness, subjective incompetence (SI), a broken sense of connection between the past and the future, as well as earlier failure re‐experiencing.^13^, ^14^ The psychiatric assistant did not recommend a plan for QTP reduction and the discontinuation of QTP and only performed supportive psychotherapy with diagnosis of demoralization. On the 29th day after hospitalization, due to edema of the face, the upper and lower extremities, and the chest wall, a new psychiatric visit was scheduled with the possibility of QTP‐induced edema. At the time, with regard to empyema associated with *Staphylococcus aureus* culture, he was thus a candidate for evacuation and thoracotomy, but this was canceled due to chest wall edema. The patient was also oriented, and not agitated. Consequently, the QTP dose was reduced to 100 mg/day according to a tapering schedule and then discontinued due to edema and the resolution of persistent delirium. As a whole, edema was temporarily relieved. Over the next 2 days, the swellings of the face and the upper extremities decreased, but the chest wall and lower extremity edema continued. In the next 4 days, the QTP was completely discontinued, and the edema finally disappeared 2 days later. Laboratory tests did not confirm the patient's edema in terms of thyroid‐stimulating hormone (TSH), albumin (Alb), electrolytes, blood urea nitrogen (BUN), liver function tests, erythrocyte sedimentation rate (ESR), and serum creatinine (CRT). Echocardiography also showed no pathological findings. The patient was not taking any other medications that could explain the edema, and he had no history of methadone‐induced edema in the past. The edema disappeared sufficiently after stopping QTP, despite methadone intake. Subsequently, the patient was discharged on the 37th day after admission. When he was discharged, he was conscious and oriented, and the wound infection was gone.

## 3 DISCUSSION

In the reported case, the absence of a medical condition responsible for edema, the time of edema development, and recovery after stopping the QTP administration in a young man supported the following hypothesis, namely a strong association between peripheral edema and the use of QTPs. According to the Naranjo Adverse Drug Reactions Probability Scale (NADRPS), the patient had a score of 7, indicating probable adverse drug reactions to QTP.^15^

There are reports of association between opium use and peripheral edema.^16^ This patient was treated with morphine for 10 days, and then, methadone was prescribed. In terms of time, this complication occurred with increasing QTP dose, and it was resolved after stopping QTP, despite the continuation of methadone.

Although the potential mechanisms of antipsychotic‐induced edema are remain speculative, previous studies suggest an association between dopaminergic antagonism and peripheral edema.^17^, ^18^ Since QTP has weak dopaminergic antagonism compared with other antipsychotic agents,^19^ other mechanisms may be thus more plausible. Therefore, some mechanisms of QTP‐induced edema phenomenon have been described. First, previous studies have shown that idiopathic edema may be related to dopaminergic antagonism, because dopamine can affect the renin–angiotensin–aldosterone system (RAAS).^20^, ^21^ Second, QTP can block α‐1 receptors, resulting in peripheral vasodilatation and edema.^22^ Third, antagonizing 5‐hydroxytryptophan 2 (5‐HTP2) receptors can also increase intracellular cyclic adenosine monophosphate (cAMP) and subsequently induce relaxation of vascular smooth muscle.^21^ Fourth, the antagonism of muscarinic 1 (M1), histaminergic 1 (H1), and 5‐HTP2 receptors can inhibit the increase in inositol triphosphate (IP3), which seems necessary for smooth muscle contraction, and leads to vasodilatation and edema.^22^ However, the exact mechanism of QTP‐induced edema, parallel to this complication in other second‐generation antipsychotics, remained unclear. An allergic reaction might also be considered as an alternative explanation for drug‐induced edema. Unfortunately, laboratory tests, including the measurement of the levels of immunoglobulin E (IgE) and complement components 3 and 4 (C3 and C4), were not performed for the patient in this regard. The unique point in this patient was the chest wall edema, which had not been reported so far, due to the lying position of this case. Two cases of facial and eyelid edema in addition to bilateral lower extremity edema had been already reported in two other studies.^9^, ^10^ The first case was a 60‐year‐old woman with major depressive disorder (MDD), who had experienced these side effects 3 days after adding a dose of 25 mg of QTP to her medication regimen, which had been resolved within a day after stopping the medication, but had relapsed once QTP had been restarted.^9^ The second case was a pateint with bilateral extremity edema accompanied by facial edema, which had been reported by Sahan et al. (2018) in Turkey. This had occurred in a 54‐year‐old woman with bipolar disorder admitted to a hospital and treated with 50 mg/mL of haloperidol decanoate and 600 mg of QTP daily. Eight days after hospitalization, she had developed bilateral lower extremity and facial edema, and 1 week after reducing the dose of QTP from 600 to 300 mg daily, the edema had been resolved.^10^ Similarly, a case report (2016) was published about lip swelling in a 65‐year‐old Italian woman with the diagnosis of refractory major depressive episode following the second administration of sustained‐release QTP at a dose of 50 mg.^23^ As well, Gopalakrishnan et al.^24^ had reported a case of bilateral pitting pedal and facial edema in a woman with no medical comorbidities after starting olanzapine treatment. The median time to onset of antipsychotic‐induced edema can therefore range from 1 day to several months after initiation of treatment.^5^ Umar and Abdullahi^25^ also found that the median age at onset for edema was 22.9 days, and the median time to resolution of edema was 10.3 days (less than 4 weeks). In most patients, the edema extended to both legs and less commonly to the hands, arms, face, eyelids, and periorbital area.^5^ In several reports, peripheral edema had improved after dose reduction of atypical antipsychotic.^26^, ^27^ Rate of antipsychotic dose escalation (gradual vs. rapid) may contribute to the development of antipsychotic‐induced edema.^5^

## 4 CONCLUSION

QTP can contribute to the development of facial and extremity, especially chest wall, edema. Although this was not a life‐threatening complication, it could postpone thoracic surgery in this patient. Therefore, it is very important to pay close attention to this complication. The main objective of this case presentation was to increase knowledge about the rare adverse effects of edema in order to avoid unnecessary investigations or relapses. Therefore, it is recommended routine screening for this adverse effect in the follow up of these patients.

## AUTHOR CONTRIBUTIONS

**Forouzan Elyasi:** Conceptualization; investigation; supervision; validation; writing – original draft; writing – review and editing. **Fatemeh Heydari:** Writing – review and editing. **KAVEH HADDADI:** Supervision; writing – review and editing.

## FUNDING INFORMATION

Not applicable.

## CONFLICT OF INTEREST STATEMENT

All authors declare no conflicts of interest.

## ETHICS STATEMENT

The research proposal was approved by the Ethics Committee affiliated in Mazandaran University of Medical Sciences [IR.MAZUMS.REC.1401.541].

## CONSENT

Written consent for the publication of this case report has been provided by the patient's father.

## Data Availability

This is a case report of a single patient. All data provided during this study are included in this article.
